# Crystal structure of undeca­potassium bis­[α-hemi­penta­hydrogen hexa­molybdoplatinate(IV)] dodeca­hydrate

**DOI:** 10.1107/S2056989015014188

**Published:** 2015-07-31

**Authors:** Hea-Chung Joo, Ki-Min Park, Uk Lee

**Affiliations:** a Department of Chemistry, Pukyong National University, 599-1 Daeyeon 3-dong, Nam-gu, Busan 608-737, Republic of Korea; b Research Institute of Natural Science, Gyeongsang National University, 501, Jinju-daero, Jinju, 660-701, Republic of Korea

**Keywords:** crystal structure, platinium(IV), platinum-containing heteropolyoxometalate, short hydrogen bond, Anderson structure heteropolyoxomolybdate

## Abstract

The title compound, K_11_[α-Pt(μ_3_-OH)_2_(μ_3_-OH_0.5_)Mo_6_(μ_3_-O)_3_(μ_2_-O)_6_O_12_]_2_·12H_2_O (simplified chemical formula K_11_[H_2.5_PtMo_6_O_24_]_2_·12H_2_O), containing the well-known Anderson-type heteropolyoxomolybdate anion, was obtained by hydro­thermal reaction at pH = *ca* 6.0. The complete polyanion dimer has 2/*m* symmetry. The locations of the H atoms with respect to protonated O atoms were obtained from difference Fourier maps, and confirmed by the inter­polyanion hydrogen bonds, bond-length elongation and bond-valence sums (BVSs). The title heteropolyanion has two types of protonated O atoms *viz.* μ_3_-OH, {Mo_2_–O(H)–Pt} and μ_3_-OH_0.5_ (disordered H atom). The [H_2.5_α-PtMo_6_O_24_]^5.5−^ polyanion forms a dimer, [(H_2.5_α-PtMo_6_O_24_)_2_]^11−^, held together by two pairs of μ_3_-O—H⋯μ_1_-O and of one disordered μ_3_-O⋯H⋯μ_3_-O hydrogen bonds. Three K^+^ ions are located on special positions (two on twofold rotation axes, one on a mirror plane), and two K^+^ ions are located on general positions with a reduced occupancy of 0.5. The remaining K^+^ ion has a reduced occupancy of 0.25 for charge balance and reasonable displacement parameters. As a result, the numbers of K^+^ and H^+^ ions in the title compound are 5.5 and 2.5, respectively.

## Abstract 2

The title heteropolyanion has two types of protonated O atoms *viz.* μ_3_-OH, {Mo_2_–O(H)–Pt} and μ_3_-OH_0.5_ (disordered H atom). The [H_2.5_α-PtMo_6_O_24_]^5.5−^ polyanion forms a dimer, [(H_2.5_α-PtMo_6_O_24_)_2_]^11−^, with 2/*m* symmetry, held together by two pairs μ_3_-O—H⋯μ_1_-O and one disordered μ_3_-O⋯H⋯μ_3_-O hydrogen bonds.

## Chemical context   

The *α* (planar structure) -*β* (bent structure) -*α* geometrical isomerization, according to stepwise protonation in the [PtMo_6_O_24_]^8−^ polyoxometalate (POM) species, *viz*. ([H_3.5_ *α*-PtMo_6_O_24_]^4.5−^ (Lee & Sasaki, 1998 ▸), [H_4_ *β*-PtMo_6_O_24_]^4−^ (Lee & Sasaki, 1998 ▸; Joo *et al.*, 1994 ▸) and [H_4.5_ *α*-PtMo_6_O_24_]^3.5−^ (Lee & Sasaki, 1998 ▸; Lee *et al.*, 2010 ▸; Joo *et al.*, 2015 ▸), is an unprecedented phenomenon in the Anderson-type heteropolyanion (Anderson, 1937 ▸) as well as in the chemistry of POMs. However, in addition, differently protonated polyanion species have been reported, *viz*. [H_2_PtMo_6_O_24_]^6−^ (Lee & Joo, 2000 ▸, 2004*b* ▸), and [H_6_PtMo_6_O_24_]^2−^ (Lee & Joo, 2006*a* ▸,*b* ▸, 2010 ▸). Less protonated than the title polyanion, the species [H_2_PtMo_6_O_24_]^6−^ was obtained in more acidic conditions (at pH 2.0 and 3.2). These polyanions are formed into dimers and polymers (in [H_6_PtMo_6_O_24_]^2−^ polyanions) by inter­polyanion hydrogen bonds. Recently, a hydrogen-bonded hexa­molybdoplatinate(IV) tetra­mer, [(PtMo_6_O_24_)_4_H_23_]^9−^, and the trimers, [(PtMo_6_O_24_)_3_H_16_]^8−^ and [(PtMo_6_O_24_)_3_H_14_]^10−^ have been reported as the tetra-*n*-butyl­ammonium and tetra-*n*-butyl­ammonium/tri­ethyl­ammonium salts, respectively (Day *et al.*, 2009 ▸). The same type of protonated species in a tungsten system, [H_2.5_PtW_6_O_24_]^5.5−^, has been reported by our group (Lee & Joo, 2004*a* ▸). We report herein the crystal structure of the title compound containing a new protonated species in the hexa­molybdoplatinate(IV) system by hemi­penta H^+^, [H_2.5_PtMo_6_O_24_]^5.5−^.

## Structural commentary   

**Figure 1:**
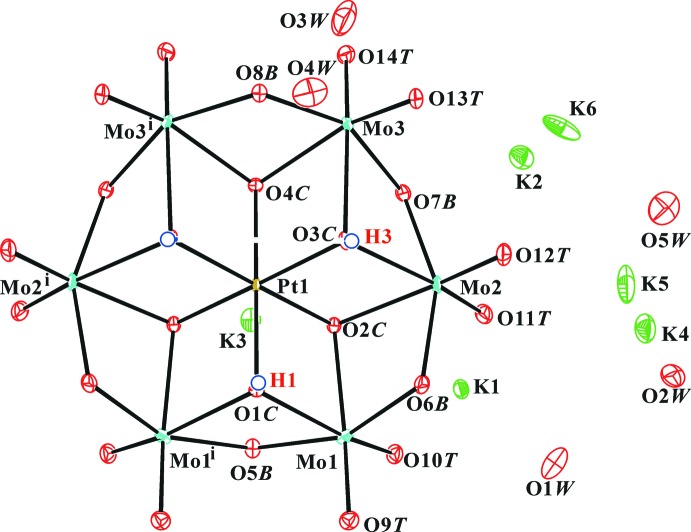
The mol­ecular structure of the [H_2.5_PtMo_6_O_24_]^5.5−^ anion and surrounding K^+^ cations and water molecules. Displacement ellipsoids are drawn at the 30% probability level. The H atoms of the polyanion are presented as small spheres of arbitrary radius and the H atoms of water mol­ecules are omitted for clarity. [Symmetry code: (i) −*x* + 1, *y*, *z*.]

**Figure 2:**
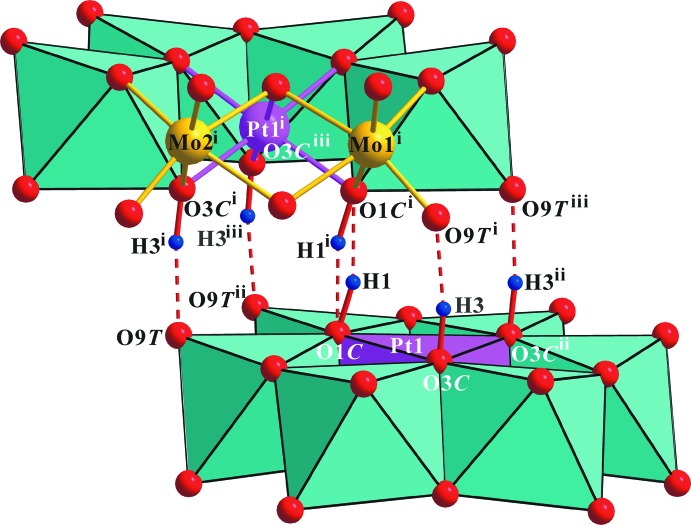
Polyhedral view of the heteropolyanion in the title compound with O—H⋯O contacts of the inter­anion hydrogen bonds shown as red dashed lines. [Symmetry codes: (i) *x*, − *y* + 1, − *z* + 1; (ii) − *x* + 1, *y*, *z*; (iii) − *x* + 1, − *y* + 1, − *z* + 1.]

Fig. 1 ▸ shows the building units of the title compound. The complete polyanion has point group symmetry *m* whereas the dimer (held together by hydrogen bonds) has 2/*m* symmetry. The O atoms of the heteropolyanion have been designated as O*T* (terminal Mo=O atom), O*B* (bridging *μ*_2_-O*B* atom; Mo—O—Mo) and O*C* (centered *μ*_3_-O atom; Mo_2-_–O*C-*–Pt). The protonated O atoms in the polyanion were confirmed by bond-valence sums (BVS; Brown & Altermatt, 1985 ▸; Brese & O’Keeffe, 1991 ▸), charge balance, bond-length elongation (Table 1 ▸) and the inter­polyanion hydrogen bonds (Fig. 2 ▸ and Table 2 ▸).

Confirmation of the protonated O atoms was strongly supported by the BVS analysis. The calculated BVSs for expected protonation atoms O1*C* and O3*C* are 1.46 and 1.39 valence units (v.u.), respectively, if the valence of the O—H bond is not included. Since the BVS value around the O atom should be 2.0 v.u., the missing valences of O1*C* and O3*C* are 0.54 and 0.61 v.u., respectively, which corresponds to the valence of the O—H bonds. The BVS value for the unproton­ated O2*C*, O4*C*, O5*B*–O8*B* atoms are 1.87, 1.78, 1.88, 1.60, 1.88 and 1.76 v.u., respectively. The value of O6*B* is relatively small but it is not protonated. As a result, the protonated O atoms are O1*C* and O3*C*.

The positions of atoms H1 and H3 on the protonated atoms O1*C* and O3*C*, respectively, were obtained from difference Fourier maps. The heteropolyanion forms a 2/*m* symmetric dimer {[H_2.5_PtMo_6_O_24_]_2_}^11−^, held together by each of the four *μ*_3_(Mo_2_Pt)-O3*C-*–H3⋯*μ*_1_(Mo)-O9*T* and one disordered *μ*_3_(Mo_2_Pt)-O1*C*⋯H1⋯*μ*_3_-O1*C* hydrogen bonds (Table 2 ▸ and Fig. 2 ▸).

While the structure of the dimeric polyanions is clear, the disorder among the potassium atoms and water mol­ecules makes it difficult to be as certain of the chemical structure in the regions in between. The K1–K3 ions were located in special positions, one on a mirror plane and two on twofold rotation axes. The calculated BVSs for the K1–K3 ions are 1.00, 0.90 and 1.00 v.u. (K^+^⋯O distance 〈 3.00 Å), respectively. Reasonable displacement parameters of K4 and K5 atoms were obtained by reducing the site occupancies to 0.5, and the BVSs for K4 and K5 ions are 1.04 and 0.91 v.u., respectively. The occupancy of K6 was further reduced to 0.25 for charge balance and reasonable displacement parameters. The calculated BVSs was 0.54 v.u. For the same reason, the occupancies of water mol­ecules O2*W*–O6*W* were reduced to 0.5. The K^+^ ions are variously coordinated by O atoms as [K1(O*T*)_6_]^+^, [K2(O*T*)_4_(O*W*)_2_]^+^, [K3(O*C*)_2_(O*B*)(O*T*)_2_(O*W*)_2_]^+^, [K4(O*T*)(O*W*)_6_]^+^, [K5(O*T*)_2_(O*W*)_3_]^+^ and [K6(O*T*)(O*W*)]^+^.

## Supra­molecular features   

The polyanion dimers are three-dimensionally linked only *via* K⋯O*B, C* and *T* inter­actions. Water mol­ecules O1*W*, O2*W* and O5*W* do not contribute to the hydrogen bonds.

## Synthesis and crystallization   

Crystals of the title compound were prepared by the reaction of K_2_MoO_4_·2H_2_O and K_2_Pt(OH)_6_ at pH = *ca* 6.0. as described in a previous report (Joo *et al.*, 1994 ▸).

## Refinement   

Crystal data, data collection and structure refinement details are summarized in Table 3 ▸. Atoms H1 and H3 in the polyanion were located in difference Fourier maps and refined with *U*_iso_(H) = 1.5*U*_eq_(O), and a distance restraint of O—H = 1.00 (3) Å using the DFIX command in *SHELXL2014/7* (Sheldrick, 2015 ▸). The occupancy of atom H1 was reduced to 0.5 because of disorder. Reasonable displacement parameters for atoms K4–K6 and water mol­ecules O2*W*–O5*W* were obtained by reducing their site occupancies to 0.5 because of disorder. The occupancy of K6 was further reduced to 0.25 for charge balance and reasonable displacement parameters. All H atoms of water mol­ecules O1*W*–O4*W* were found in difference Fourier maps and refined with distance and angle restraints of O—H = 0.85 (3) Å and H*A*—O*W*—H*B* = 1.35 (3) Å, respectively, using the command DFIX, and were included in the refinement with *U*_iso_(H) = 1.5*U*_eq_(O). The H atoms on O5*W* were positioned geometrically and refined using a riding model (HFIX 23), with O*W*—H = 0.97 Å and *U*_iso_(H) = 1.5*U*_eq_(O).

The reflections (0,2,0) and (3,1,2) were omitted in the final refinement as they were obscured by the beamstop. The highest peak in the difference map is 0.86 Å from Pt1 and the deepest hole is 0.88 Å from Pt1.

## Supplementary Material

Crystal structure: contains datablock(s) New_Global_Publ_Block, I. DOI: 10.1107/S2056989015014188/br2251sup1.cif

CCDC reference: 1415450

Additional supporting information:  crystallographic information; 3D view; checkCIF report

## Figures and Tables

**Table 1:** Selected geometric parameters (, )

Pt1O1*C*	2.003(5)	Mo1O2*C*	2.110(4)
Pt1O2*C*	1.981(3)	Mo2O2*C*	2.141(3)
Pt1O3*C*	2.000(3)	Mo2O3*C*	2.307(4)
Pt1O4*C*	2.001(5)	Mo3O3*C*	2.316(4)
Mo1O1*C*	2.277(3)	Mo3O4*C*	2.140(3)
			
Pt1O1*C*Mo1	98.38(17)	Pt1O3*C*Mo2	99.35(15)
Mo1O1*C*Mo1^i^	92.86(18)	Pt1O3*C*Mo3	99.91(14)
Pt1O2*C*Mo1	104.91(15)	Mo2O3*C*Mo3	92.10(13)
Pt1O2*C*Mo2	105.82(15)	Pt1O4*C*Mo3	106.09(17)
Mo1O2*C*Mo2	99.12(14)	Mo3O4*C*Mo3^i^	97.5(2)

Symmetry code: (i) 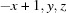.

**Table 2:** Hydrogen-bond geometry (, )

*D*H*A*	*D*H	H*A*	*D* *A*	*D*H*A*
O1*C*H1O1*C* ^ii^	0.99(3)	1.62(6)	2.595(10)	165(17)
O3*C*H3O9*T* ^iii^	0.97(3)	1.64(3)	2.605(5)	171(5)
O3*W*H3*B*O4*W* ^i^	0.85(3)	2.07(14)	2.697(15)	130(15)
O4*W*H4*A*O9*T* ^iii^	0.85(3)	2.06(7)	2.826(11)	150(12)
O4*W*H4*B*O3*W* ^i^	0.85(3)	1.89(5)	2.697(15)	159(5)

Symmetry codes: (i) 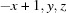; (ii) 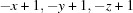; (iii) 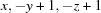.

**Table 3:** Experimental details

Crystal data	
Chemical formula	K_11_[H_2.5_PtMo_6_O_24_]_2_12H_2_O
*M*_r_	1480.40
Crystal system, space group	Orthorhombic, *C* *m* *c* *e*
Temperature (K)	173
*a*, *b*, *c* ()	16.8552(4), 22.7112(7), 15.5503(4)
*V* (^3^)	5952.7(3)
*Z*	8
Radiation type	Mo *K*
(mm^1^)	8.00
Crystal size (mm)	0.25 0.15 0.14

Data collection	
Diffractometer	Bruker *SMART* APEXII CCD
Absorption correction	Multi-scan (*SADABS*; Bruker, 2009 ▸)
*T*_min_, *T*_max_	0.424, 0.746
No. of measured, independent and observed [*I* > 2(*I*)] reflections	16741, 3826, 3257
*R*_int_	0.044
(sin /)_max_ (^1^)	0.667

Refinement	
*R*[*F* ^2^ > 2(*F* ^2^)], *wR*(*F* ^2^), *S*	0.031, 0.076, 1.06
No. of reflections	3826
No. of parameters	264
No. of restraints	14
H-atom treatment	H atoms treated by a mixture of independent and constrained refinement
_max_, _min_ (e ^3^)	1.50, 1.32

Computer programs: *APEX2* and *SAINT* (Bruker, 2009 ▸), *SHELXS2014* (Sheldrick, 2008 ▸), *SHELXL2014* (Sheldrick, 2015 ▸), *ORTEP-3 for Windows* (Farrugia, 2012 ▸) and *DIAMOND* (Brandenburg, 1998 ▸).

## supplementary crystallographic information

### Crystal data

**Table:**

K_11_[H_2.5_PtMo_6_O_24_]_2_·12H_2_O	*D*_x_ = 3.304 Mg m^−^^3^
*M**_r_* = 1480.40	Mo *K*α radiation, λ = 0.71073 Å
Orthorhombic, *C**m**c**e*	Cell parameters from 3642 reflections
*a* = 16.8552 (4) Å	θ = 2.4–28.2°
*b* = 22.7112 (7) Å	µ = 8.00 mm^−^^1^
*c* = 15.5503 (4) Å	*T* = 173 K
*V* = 5952.7 (3) Å^3^	Block, pale yellow
*Z* = 8	0.25 × 0.15 × 0.14 mm
*F*(000) = 5512	

### Data collection

**Table:**

Bruker SMART APEXII CCD diffractometer	3826 independent reflections
Radiation source: rotating anode	3257 reflections with *I* > 2σ(*I*)
Detector resolution: 10.0 pixels mm^-1^	*R*_int_ = 0.044
φ and ω scans	θ_max_ = 28.3°, θ_min_ = 2.0°
Absorption correction: multi-scan (*SADABS*; Bruker, 2009)	*h* = −22→22
*T*_min_ = 0.424, *T*_max_ = 0.746	*k* = −30→17
16741 measured reflections	*l* = −16→20

### Refinement

**Table:**

Refinement on *F*^2^	Primary atom site location: structure-invariant direct methods
Least-squares matrix: full	Secondary atom site location: difference Fourier map
*R*[*F*^2^ > 2σ(*F*^2^)] = 0.031	Hydrogen site location: difference Fourier map
*wR*(*F*^2^) = 0.076	H atoms treated by a mixture of independent and constrained refinement
*S* = 1.06	*w* = 1/[σ^2^(*F*_o_^2^) + (0.0297*P*)^2^ + 46.2991*P*] where *P* = (*F*_o_^2^ + 2*F*_c_^2^)/3
3826 reflections	(Δ/σ)_max_ = 0.001
264 parameters	Δρ_max_ = 1.50 e Å^−^^3^
14 restraints	Δρ_min_ = −1.32 e Å^−^^3^

### Special details

**Table:**

Geometry. All esds (except the esd in the dihedral angle between two l.s. planes) are estimated using the full covariance matrix. The cell esds are taken into account individually in the estimation of esds in distances, angles and torsion angles; correlations between esds in cell parameters are only used when they are defined by crystal symmetry. An approximate (isotropic) treatment of cell esds is used for estimating esds involving l.s. planes.

### Fractional atomic coordinates and isotropic or equivalent isotropic displacement parameters (Å^2^)

**Table:**

	*x*	*y*	*z*	*U*_iso_*/*U*_eq_	Occ. (<1)
Pt1	0.5000	0.37721 (2)	0.50594 (2)	0.00882 (8)	
Mo1	0.40210 (3)	0.43798 (2)	0.34971 (3)	0.01424 (11)	
Mo2	0.30535 (3)	0.36824 (2)	0.49776 (3)	0.01495 (11)	
Mo3	0.40452 (2)	0.29903 (2)	0.65278 (3)	0.01481 (11)	
O1C	0.5000	0.4557 (2)	0.4473 (3)	0.0123 (10)	
H1	0.5000	0.485 (6)	0.495 (8)	0.018*	0.5
O2C	0.41239 (19)	0.36081 (16)	0.4244 (2)	0.0125 (7)	
O3C	0.41006 (19)	0.39059 (16)	0.5875 (2)	0.0131 (7)	
H3	0.402 (3)	0.4250 (19)	0.623 (3)	0.020*	
O4C	0.5000	0.2979 (2)	0.5621 (3)	0.0131 (10)	
O5B	0.5000	0.4138 (2)	0.2959 (3)	0.0160 (11)	
O6B	0.3265 (2)	0.44740 (18)	0.4460 (2)	0.0208 (8)	
O7B	0.3379 (2)	0.29588 (17)	0.5512 (2)	0.0176 (8)	
O8B	0.5000	0.3212 (2)	0.7162 (3)	0.0158 (11)	
O9T	0.4015 (2)	0.51246 (17)	0.3235 (3)	0.0187 (8)	
O10T	0.3402 (2)	0.40612 (17)	0.2754 (2)	0.0196 (8)	
O11T	0.2431 (2)	0.3418 (2)	0.4200 (3)	0.0266 (9)	
O12T	0.2448 (2)	0.3962 (2)	0.5763 (3)	0.0273 (10)	
O13T	0.3397 (2)	0.33250 (19)	0.7226 (3)	0.0237 (9)	
O14T	0.4017 (2)	0.22555 (18)	0.6778 (3)	0.0228 (9)	
K1	0.2500	0.30728 (8)	0.2500	0.0221 (4)	
K2	0.2500	0.43286 (8)	0.7500	0.0267 (4)	
K3	0.5000	0.28050 (8)	0.33118 (12)	0.0254 (4)	
K4	0.09783 (17)	0.43827 (17)	0.5206 (2)	0.0371 (7)	0.5
K5	0.09202 (17)	0.3486 (2)	0.4765 (2)	0.0595 (12)	0.5
K6	0.1660 (5)	0.2717 (3)	0.6235 (4)	0.0478 (19)	0.25
O1W	0.1839 (3)	0.4814 (2)	0.3701 (4)	0.0412 (13)	
H1A	0.159 (4)	0.451 (2)	0.346 (5)	0.062*	
H1B	0.219 (4)	0.465 (3)	0.402 (4)	0.062*	
O2W	0.0345 (5)	0.3851 (4)	0.3785 (6)	0.033 (2)	0.5
H2A	0.066 (6)	0.355 (3)	0.371 (10)	0.049*	0.5
H2B	0.065 (6)	0.414 (4)	0.363 (10)	0.049*	0.5
O3W	0.4326 (6)	0.4488 (5)	0.9311 (7)	0.041 (3)	0.5
H3A	0.466 (7)	0.470 (6)	0.958 (7)	0.062*	0.5
H3B	0.446 (9)	0.452 (7)	0.878 (3)	0.062*	0.5
O4W	0.4571 (6)	0.4175 (4)	0.8141 (6)	0.038 (2)	0.5
H4A	0.448 (8)	0.448 (4)	0.785 (8)	0.057*	0.5
H4B	0.5000	0.424 (5)	0.842 (4)	0.057*	
O5W	0.0461 (6)	0.2998 (5)	0.5369 (7)	0.051 (3)	0.5
H5A	0.0637	0.2610	0.5192	0.077*	0.5
H5B	0.0637	0.3054	0.5958	0.077*	0.5

### Atomic displacement parameters (Å^2^)

**Table:**

	*U*^11^	*U*^22^	*U*^33^	*U*^12^	*U*^13^	*U*^23^
Pt1	0.00455 (12)	0.00998 (13)	0.01192 (13)	0.000	0.000	−0.00001 (10)
Mo1	0.0159 (2)	0.0116 (2)	0.0152 (2)	0.00145 (17)	−0.00367 (16)	0.00085 (17)
Mo2	0.0058 (2)	0.0224 (3)	0.0166 (2)	−0.00089 (16)	0.00046 (15)	0.00087 (18)
Mo3	0.0083 (2)	0.0173 (2)	0.0188 (2)	−0.00036 (17)	0.00244 (16)	0.00565 (18)
O1C	0.013 (2)	0.011 (2)	0.014 (2)	0.000	0.000	0.0032 (19)
O2C	0.0108 (17)	0.0133 (18)	0.0134 (17)	−0.0018 (14)	−0.0018 (13)	−0.0013 (14)
O3C	0.0082 (16)	0.0160 (19)	0.0151 (17)	0.0009 (14)	0.0038 (13)	−0.0016 (14)
O4C	0.012 (2)	0.010 (2)	0.017 (3)	0.000	0.000	0.003 (2)
O5B	0.014 (2)	0.017 (3)	0.018 (3)	0.000	0.000	−0.005 (2)
O6B	0.0136 (18)	0.029 (2)	0.0201 (19)	0.0078 (17)	0.0040 (15)	0.0056 (17)
O7B	0.0113 (18)	0.021 (2)	0.0211 (19)	−0.0064 (15)	−0.0024 (14)	0.0052 (16)
O8B	0.012 (2)	0.018 (3)	0.017 (3)	0.000	0.000	0.001 (2)
O9T	0.020 (2)	0.0154 (19)	0.021 (2)	−0.0003 (15)	−0.0019 (16)	0.0000 (15)
O10T	0.0150 (19)	0.022 (2)	0.0214 (19)	−0.0014 (16)	−0.0032 (15)	−0.0007 (16)
O11T	0.0155 (19)	0.045 (3)	0.020 (2)	−0.0050 (19)	−0.0028 (15)	0.0079 (19)
O12T	0.0132 (19)	0.046 (3)	0.023 (2)	0.0072 (18)	0.0036 (16)	0.006 (2)
O13T	0.0104 (18)	0.037 (3)	0.024 (2)	0.0049 (17)	0.0041 (15)	0.0083 (18)
O14T	0.0162 (19)	0.025 (2)	0.028 (2)	−0.0049 (16)	−0.0038 (16)	0.0080 (18)
K1	0.0160 (8)	0.0282 (10)	0.0223 (9)	0.000	0.0027 (6)	0.000
K2	0.0275 (10)	0.0299 (10)	0.0227 (9)	0.000	0.0033 (7)	0.000
K3	0.0253 (9)	0.0248 (10)	0.0262 (9)	0.000	0.000	−0.0107 (7)
K4	0.0198 (14)	0.061 (2)	0.0307 (16)	0.0001 (13)	−0.0029 (12)	−0.0010 (15)
K5	0.0178 (15)	0.118 (4)	0.0425 (19)	0.0099 (18)	0.0007 (13)	−0.013 (2)
K6	0.091 (5)	0.019 (3)	0.034 (3)	−0.010 (3)	0.037 (3)	−0.007 (2)
O1W	0.038 (3)	0.039 (3)	0.047 (3)	0.016 (2)	−0.017 (2)	−0.010 (2)
O2W	0.039 (5)	0.037 (5)	0.022 (4)	0.006 (4)	0.007 (4)	0.000 (4)
O3W	0.024 (5)	0.067 (8)	0.032 (6)	0.010 (5)	−0.009 (5)	−0.005 (5)
O4W	0.052 (6)	0.027 (5)	0.035 (5)	0.004 (5)	−0.002 (4)	0.001 (4)
O5W	0.059 (7)	0.049 (7)	0.045 (6)	0.016 (6)	−0.003 (5)	−0.021 (5)

### Geometric parameters (Å, º)

**Table:**

Pt1—O1C	2.003 (5)	O13T—K2	2.768 (4)
Pt1—O2C	1.981 (3)	O14T—K1^ii^	2.890 (4)
Pt1—O3C	2.000 (3)	O14T—K3^iii^	2.907 (4)
Pt1—O4C	2.001 (5)	O14T—K5^ii^	2.934 (6)
Mo1—O1C	2.277 (3)	K1—O10T^vi^	2.740 (4)
Mo1—O2C	2.110 (4)	K1—O11T^vi^	2.760 (4)
Mo2—O2C	2.141 (3)	K1—O14T^ii^	2.890 (4)
Mo2—O3C	2.307 (4)	K1—O14T^vii^	2.890 (4)
Mo3—O3C	2.316 (4)	K2—O13T^v^	2.768 (4)
Mo3—O4C	2.140 (3)	K2—O12T^v^	2.828 (4)
Pt1—O2C^i^	1.981 (3)	K2—O1W^viii^	2.919 (6)
Pt1—O3C^i^	2.000 (3)	K2—O1W^ix^	2.919 (6)
Pt1—K3	3.4943 (18)	K2—O9T^viii^	3.060 (4)
Mo1—O10T	1.717 (4)	K2—O9T^ix^	3.060 (4)
Mo1—O9T	1.740 (4)	K3—O2C^i^	2.759 (4)
Mo1—O5B	1.930 (3)	K3—O5W^x^	2.852 (12)
Mo1—O6B	1.978 (4)	K3—O5W^ii^	2.852 (12)
Mo1—K3	3.9494 (17)	K3—O14T^vii^	2.907 (4)
Mo2—O11T	1.709 (4)	K3—O14T^xi^	2.907 (4)
Mo2—O12T	1.714 (4)	K3—O8B^xi^	2.921 (5)
Mo2—O7B	1.921 (4)	K4—O3W^v^	0.940 (11)
Mo2—O6B	2.002 (4)	K4—O2W	2.735 (10)
Mo3—O14T	1.714 (4)	K4—O4W^v^	2.773 (11)
Mo3—O13T	1.717 (4)	K4—O1W^ix^	2.884 (6)
Mo3—O7B	1.939 (4)	K4—O3W^xii^	2.894 (10)
Mo3—O8B	1.953 (3)	K4—O1W	2.923 (7)
Mo3—Mo3^i^	3.2187 (8)	K4—O3W^iv^	2.963 (12)
O1C—Mo1^i^	2.278 (3)	K4—O5W	3.274 (12)
O1C—H1	0.99 (3)	K5—O5W	1.647 (13)
O2C—K3	2.759 (4)	K5—O2W	1.987 (10)
O2C—K6^ii^	3.370 (7)	K5—O3W^v^	2.723 (13)
O3C—H3	0.97 (3)	K5—O5W^xiii^	2.744 (11)
O4C—Mo3^i^	2.140 (3)	K5—O2W^xiii^	2.750 (9)
O5B—Mo1^i^	1.930 (3)	K5—O14T^ii^	2.934 (6)
O5B—K3	3.077 (6)	K6—O5W	2.512 (14)
O7B—K6^ii^	3.121 (7)	K6—O13T^v^	2.765 (7)
O7B—K6	3.155 (8)	K6—O11T^ii^	3.074 (8)
O8B—Mo3^i^	1.953 (3)	K6—O7B^ii^	3.121 (7)
O8B—K3^iii^	2.921 (5)	K6—O2C^ii^	3.370 (7)
O9T—K2^iv^	3.060 (4)	O1W—H1A	0.88 (3)
O10T—K1	2.740 (4)	O1W—H1B	0.86 (3)
O11T—K5	2.698 (5)	O2W—H2A	0.86 (3)
O11T—K1	2.760 (4)	O2W—H2B	0.87 (3)
O11T—K6^ii^	3.074 (8)	O3W—H3A	0.86 (3)
O12T—K4	2.792 (5)	O3W—H3B	0.85 (3)
O12T—K2	2.828 (4)	O4W—H4A	0.85 (3)
O12T—K5	3.195 (6)	O4W—H4B	0.85 (3)
O12T—K6	3.210 (7)	O5W—H5A	0.9700
O13T—K6^v^	2.765 (7)	O5W—H5B	0.9700
			
Pt1—O1C—Mo1	98.38 (17)	O2C—K3—O5W^x^	101.0 (2)
Mo1—O1C—Mo1^i^	92.86 (18)	O2C^i^—K3—O5W^x^	84.2 (2)
Pt1—O2C—Mo1	104.91 (15)	O2C—K3—O5W^ii^	84.2 (2)
Pt1—O2C—Mo2	105.82 (15)	O2C^i^—K3—O5W^ii^	101.0 (2)
Mo1—O2C—Mo2	99.12 (14)	O5W^x^—K3—O5W^ii^	31.6 (4)
Pt1—O3C—Mo2	99.35 (15)	O2C—K3—O14T^vii^	99.06 (11)
Pt1—O3C—Mo3	99.91 (14)	O2C^i^—K3—O14T^vii^	140.13 (13)
Mo2—O3C—Mo3	92.10 (13)	O5W^x^—K3—O14T^vii^	135.6 (2)
Pt1—O4C—Mo3	106.09 (17)	O5W^ii^—K3—O14T^vii^	113.9 (2)
Mo3—O4C—Mo3^i^	97.5 (2)	O2C—K3—O14T^xi^	140.14 (13)
O2C^i^—Pt1—O2C	96.4 (2)	O2C^i^—K3—O14T^xi^	99.06 (11)
O2C^i^—Pt1—O3C	177.79 (14)	O5W^x^—K3—O14T^xi^	113.9 (2)
O2C—Pt1—O3C	82.50 (15)	O5W^ii^—K3—O14T^xi^	135.6 (2)
O2C^i^—Pt1—O3C^i^	82.50 (15)	O14T^vii^—K3—O14T^xi^	69.49 (16)
O2C—Pt1—O3C^i^	177.79 (14)	O2C—K3—O8B^xi^	147.63 (7)
O3C—Pt1—O3C^i^	98.6 (2)	O2C^i^—K3—O8B^xi^	147.63 (7)
O2C^i^—Pt1—O4C	96.31 (14)	O5W^x^—K3—O8B^xi^	86.3 (2)
O2C—Pt1—O4C	96.31 (14)	O5W^ii^—K3—O8B^xi^	86.3 (2)
O3C—Pt1—O4C	81.95 (14)	O14T^vii^—K3—O8B^xi^	57.34 (11)
O3C^i^—Pt1—O4C	81.94 (14)	O14T^xi^—K3—O8B^xi^	57.34 (11)
O2C^i^—Pt1—O1C	82.88 (14)	O2C—K3—O5B	56.17 (11)
O2C—Pt1—O1C	82.88 (14)	O2C^i^—K3—O5B	56.16 (11)
O3C—Pt1—O1C	98.84 (14)	O5W^x^—K3—O5B	139.2 (2)
O3C^i^—Pt1—O1C	98.84 (14)	O5W^ii^—K3—O5B	139.2 (2)
O4C—Pt1—O1C	178.8 (2)	O14T^vii^—K3—O5B	84.27 (12)
O10T—Mo1—O9T	104.34 (18)	O14T^xi^—K3—O5B	84.27 (12)
O10T—Mo1—O5B	96.18 (18)	O8B^xi^—K3—O5B	131.98 (15)
O9T—Mo1—O5B	100.4 (2)	O3W^v^—K4—O2W	123.0 (7)
O10T—Mo1—O6B	99.42 (17)	O3W^v^—K4—O4W^v^	28.4 (7)
O9T—Mo1—O6B	93.92 (17)	O2W—K4—O4W^v^	122.9 (3)
O5B—Mo1—O6B	155.52 (18)	O3W^v^—K4—O12T	108.9 (6)
O10T—Mo1—O2C	94.06 (16)	O2W—K4—O12T	116.4 (2)
O9T—Mo1—O2C	159.58 (16)	O4W^v^—K4—O12T	87.2 (2)
O5B—Mo1—O2C	86.13 (18)	O3W^v^—K4—O1W^ix^	69.1 (7)
O6B—Mo1—O2C	74.10 (15)	O2W—K4—O1W^ix^	162.1 (3)
O10T—Mo1—O1C	164.43 (18)	O4W^v^—K4—O1W^ix^	74.3 (3)
O9T—Mo1—O1C	89.35 (18)	O12T—K4—O1W^ix^	65.64 (16)
O5B—Mo1—O1C	73.73 (16)	O3W^v^—K4—O3W^xii^	41.1 (6)
O6B—Mo1—O1C	86.76 (15)	O2W—K4—O3W^xii^	82.6 (3)
O2C—Mo1—O1C	73.78 (15)	O4W^v^—K4—O3W^xii^	56.8 (3)
O11T—Mo2—O12T	105.6 (2)	O12T—K4—O3W^xii^	143.3 (2)
O11T—Mo2—O7B	100.41 (18)	O1W^ix^—K4—O3W^xii^	106.2 (3)
O12T—Mo2—O7B	100.32 (18)	O3W^v^—K4—O1W	145.6 (8)
O11T—Mo2—O6B	98.06 (18)	O2W—K4—O1W	72.2 (2)
O12T—Mo2—O6B	93.40 (19)	O4W^v^—K4—O1W	164.8 (3)
O7B—Mo2—O6B	153.03 (15)	O12T—K4—O1W	85.57 (15)
O11T—Mo2—O2C	96.48 (16)	O1W^ix^—K4—O1W	90.59 (19)
O12T—Mo2—O2C	155.60 (17)	O3W^xii^—K4—O1W	131.1 (3)
O7B—Mo2—O2C	85.55 (15)	O3W^v^—K4—O3W^iv^	93.5 (8)
O6B—Mo2—O2C	72.94 (14)	O2W—K4—O3W^iv^	86.3 (3)
O11T—Mo2—O3C	167.10 (17)	O4W^v^—K4—O3W^iv^	121.7 (3)
O12T—Mo2—O3C	86.74 (16)	O12T—K4—O3W^iv^	126.5 (2)
O7B—Mo2—O3C	73.03 (14)	O1W^ix^—K4—O3W^iv^	79.4 (3)
O6B—Mo2—O3C	84.81 (14)	O3W^xii^—K4—O3W^iv^	83.3 (3)
O2C—Mo2—O3C	72.23 (13)	O1W—K4—O3W^iv^	54.5 (2)
O14T—Mo3—O13T	105.6 (2)	O3W^v^—K4—O5W	92.1 (8)
O14T—Mo3—O7B	97.66 (17)	O2W—K4—O5W	62.3 (3)
O13T—Mo3—O7B	99.35 (17)	O4W^v^—K4—O5W	71.1 (3)
O14T—Mo3—O8B	99.1 (2)	O12T—K4—O5W	83.3 (2)
O13T—Mo3—O8B	95.24 (18)	O1W^ix^—K4—O5W	134.1 (2)
O7B—Mo3—O8B	153.87 (17)	O3W^xii^—K4—O5W	78.6 (3)
O14T—Mo3—O4C	99.17 (19)	O1W—K4—O5W	121.1 (2)
O13T—Mo3—O4C	154.17 (19)	O3W^iv^—K4—O5W	145.3 (3)
O7B—Mo3—O4C	84.17 (15)	O5W—K5—O11T	126.2 (4)
O8B—Mo3—O4C	73.51 (16)	O2W—K5—O11T	103.6 (3)
O14T—Mo3—O3C	167.13 (17)	O5W—K5—O3W^v^	100.9 (4)
O13T—Mo3—O3C	84.56 (16)	O2W—K5—O3W^v^	89.0 (4)
O7B—Mo3—O3C	72.52 (14)	O11T—K5—O3W^v^	111.3 (3)
O8B—Mo3—O3C	87.51 (17)	O5W—K5—O5W^xiii^	30.0 (4)
O4C—Mo3—O3C	72.02 (15)	O2W—K5—O5W^xiii^	91.0 (4)
Pt1—O1C—Mo1^i^	98.38 (17)	O11T—K5—O5W^xiii^	152.7 (3)
Mo1—O5B—Mo1^i^	117.5 (3)	O3W^v^—K5—O5W^xiii^	91.6 (3)
Mo1—O6B—Mo2	108.78 (18)	O5W—K5—O2W^xiii^	98.9 (4)
Mo2—O7B—Mo3	119.1 (2)	O2W—K5—O2W^xiii^	21.7 (3)
Mo3^i^—O8B—Mo3	111.0 (2)	O11T—K5—O2W^xiii^	124.7 (3)
O10T—K1—O10T^vi^	69.96 (16)	O3W^v^—K5—O2W^xiii^	85.5 (3)
O10T—K1—O11T^vi^	83.21 (13)	O5W^xiii^—K5—O2W^xiii^	69.7 (3)
O10T^vi^—K1—O11T^vi^	69.68 (12)	O5W—K5—O14T^ii^	95.6 (4)
O10T—K1—O11T	69.68 (12)	O2W—K5—O14T^ii^	68.3 (3)
O10T^vi^—K1—O11T	83.21 (13)	O11T—K5—O14T^ii^	70.56 (14)
O11T^vi^—K1—O11T	147.0 (2)	O3W^v^—K5—O14T^ii^	156.5 (3)
O10T—K1—O14T^ii^	130.28 (12)	O5W^xiii^—K5—O14T^ii^	94.5 (3)
O10T^vi^—K1—O14T^ii^	77.07 (11)	O2W^xiii^—K5—O14T^ii^	75.4 (2)
O11T^vi^—K1—O14T^ii^	118.84 (11)	O5W—K5—K5^xiii^	62.0 (4)
O11T—K1—O14T^ii^	70.39 (12)	O2W—K5—K5^xiii^	60.8 (3)
O10T—K1—O14T^vii^	77.07 (11)	O5W—K5—O12T	109.2 (4)
O10T^vi^—K1—O14T^vii^	130.28 (12)	O2W—K5—O12T	128.6 (4)
O11T^vi^—K1—O14T^vii^	70.39 (12)	O11T—K5—O12T	54.31 (13)
O11T—K1—O14T^vii^	118.84 (11)	O3W^v^—K5—O12T	65.4 (2)
O14T^ii^—K1—O14T^vii^	150.10 (18)	O5W^xiii^—K5—O12T	130.8 (3)
O13T—K2—O13T^v^	69.14 (16)	O2W^xiii^—K5—O12T	142.4 (3)
O13T—K2—O12T	68.14 (12)	O14T^ii^—K5—O12T	124.24 (14)
O13T^v^—K2—O12T	83.57 (13)	O5W—K6—O13T^v^	108.0 (3)
O13T—K2—O12T^v^	83.57 (13)	O5W—K6—O11T^ii^	119.7 (3)
O13T^v^—K2—O12T^v^	68.14 (12)	O13T^v^—K6—O11T^ii^	128.8 (3)
O12T—K2—O12T^v^	145.8 (2)	O5W—K6—O7B^ii^	69.0 (3)
O13T—K2—O1W^viii^	116.05 (13)	O13T^v^—K6—O7B^ii^	176.7 (3)
O13T^v^—K2—O1W^viii^	131.23 (13)	O11T^ii^—K6—O7B^ii^	53.59 (14)
O12T—K2—O1W^viii^	145.01 (14)	O5W—K6—O7B	120.2 (3)
O12T^v^—K2—O1W^viii^	64.73 (13)	O13T^v^—K6—O7B	104.7 (3)
O13T—K2—O1W^ix^	131.23 (13)	O11T^ii^—K6—O7B	67.06 (18)
O13T^v^—K2—O1W^ix^	116.05 (13)	O7B^ii^—K6—O7B	78.15 (17)
O12T—K2—O1W^ix^	64.73 (13)	O5W—K6—O12T	89.2 (3)
O12T^v^—K2—O1W^ix^	145.01 (14)	O13T^v^—K6—O12T	76.85 (18)
O1W^viii^—K2—O1W^ix^	96.3 (2)	O11T^ii^—K6—O12T	118.9 (2)
O13T—K2—O9T^viii^	147.91 (12)	O7B^ii^—K6—O12T	104.03 (19)
O13T^v^—K2—O9T^viii^	79.69 (11)	O7B—K6—O12T	52.07 (14)
O12T—K2—O9T^viii^	116.73 (11)	O5W—K6—O2C^ii^	78.0 (3)
O12T^v^—K2—O9T^viii^	77.81 (11)	O13T^v^—K6—O2C^ii^	128.6 (2)
O1W^viii^—K2—O9T^viii^	79.00 (12)	O11T^ii^—K6—O2C^ii^	52.97 (13)
O1W^ix^—K2—O9T^viii^	69.48 (13)	O7B^ii^—K6—O2C^ii^	50.21 (13)
O13T—K2—O9T^ix^	79.69 (11)	O7B—K6—O2C^ii^	115.9 (2)
O13T^v^—K2—O9T^ix^	147.92 (12)	O12T—K6—O2C^ii^	153.9 (2)
O12T—K2—O9T^ix^	77.81 (11)	H1A—O1W—H1B	103 (4)
O12T^v^—K2—O9T^ix^	116.74 (11)	H2A—O2W—H2B	101 (5)
O1W^viii^—K2—O9T^ix^	69.48 (13)	H3A—O3W—H3B	105 (5)
O1W^ix^—K2—O9T^ix^	79.00 (12)	H4A—O4W—H4B	107 (5)
O9T^viii^—K2—O9T^ix^	132.12 (16)	H5A—O5W—H5B	107.1
O2C—K3—O2C^i^	64.72 (14)		

Symmetry codes: (i) −*x*+1, *y*, *z*; (ii) −*x*+1/2, −*y*+1/2, −*z*+1; (iii) −*x*+1, −*y*+1/2, *z*+1/2; (iv) −*x*+1/2, −*y*+1, *z*−1/2; (v) −*x*+1/2, *y*, −*z*+3/2; (vi) −*x*+1/2, *y*, −*z*+1/2; (vii) *x*, −*y*+1/2, *z*−1/2; (viii) −*x*+1/2, −*y*+1, *z*+1/2; (ix) *x*, −*y*+1, −*z*+1; (x) *x*+1/2, −*y*+1/2, −*z*+1; (xi) −*x*+1, −*y*+1/2, *z*−1/2; (xii) *x*−1/2, *y*, −*z*+3/2; (xiii) −*x*, *y*, *z*.

### Hydrogen-bond geometry (Å, º)

**Table:**

*D*—H···*A*	*D*—H	H···*A*	*D*···*A*	*D*—H···*A*
O1*C*—H1···O1*C*^xiv^	0.99 (3)	1.62 (6)	2.595 (10)	165 (17)
O3*C*—H3···O9*T*^ix^	0.97 (3)	1.64 (3)	2.605 (5)	171 (5)
O3*W*—H3*B*···O4*W*^i^	0.85 (3)	2.07 (14)	2.697 (15)	130 (15)
O4*W*—H4*A*···O9*T*^ix^	0.85 (3)	2.06 (7)	2.826 (11)	150 (12)
O4*W*—H4*B*···O3*W*^i^	0.85 (3)	1.89 (5)	2.697 (15)	159 (5)

Symmetry codes: (i) −*x*+1, *y*, *z*; (ix) *x*, −*y*+1, −*z*+1; (xiv) −*x*+1, −*y*+1, −*z*+1.
